# Distinctive Human Dynamics of Semantic Uncertainty: Contextual Bias Accelerates Lexical Disambiguation

**DOI:** 10.3390/bs15091159

**Published:** 2025-08-26

**Authors:** Yang Lei, Linyan Liu, Jie Chen, Chan Tang, Siyi Fan, Yongqiang Cai, Guosheng Ding

**Affiliations:** 1 State Key Laboratory of Cognitive Neuroscience and Learning & IDG/McGovern Institute for Brain Research, Beijing Normal University, Beijing 100875, China; songfenglandu@outlook.com (Y.L.);; 2 School of Mathematical Sciences, Laboratory of Mathematics and Complex Systems, Ministry of Education, Beijing Normal University, Beijing 100875, China

**Keywords:** lexical disambiguation, uncertainty, eye-tracking, LLM

## Abstract

This study investigated the dynamic resolution of lexical–semantic ambiguity during sentence comprehension, focusing on how uncertainty evolves as contextual information accumulates. Using time-resolved eye-tracking and a novel entropy-based measure derived from group-level semantic choice distributions, we quantified semantic uncertainty at a fine-grained temporal resolution for ambiguous words. By parametrically manipulating the semantic bias strength of the sentence context, we examined how context guides disambiguation over time. The results showed that semantic uncertainty declined gradually over temporal segments and dropped sharply following the onset of ambiguous words, reflecting both incremental integration and syntactic anchoring. A stronger contextual bias led to faster reductions in uncertainty, with effects following a near-linear trend. These findings support dynamic semantic processing models that assume continuous, context-sensitive convergence toward intended meanings. In contrast, a pretrained Chinese BERT model (RoBERTa-wwm-ext) showed similar overall trends in uncertainty reduction but lacked sensitivity to contextual bias. This discrepancy suggests that, while language models can approximate human-level disambiguation broadly, they fail to capture fine-grained semantic modulation driven by context. These findings provide a novel empirical characterization of disambiguation dynamics and offer a new methodological approach to capturing real-time semantic uncertainty. The observed divergence between human and model performance may inform future improvements to language models and contributes to our understanding of possible architectural differences between human and artificial semantic systems.

## 1. Introduction

Semantic ambiguity is a pervasive feature of human language (Piantadosi et al., 2012), and ambiguous words in particular pose a substantial challenge to language comprehension. For example, approximately 80% of common English words possess multiple senses (J. Rodd et al., 2002). However, misunderstandings rarely occur in everyday communication, largely because ambiguous words are typically embedded in rich contextual environments that enable listeners or readers to infer the intended meaning based on surrounding information (Duffy et al., 1988). This disambiguation process is not instantaneous; rather, it unfolds incrementally as the semantic representation evolves over time. Language users progressively narrow down the space of possible meanings as linguistic input accumulates, ultimately arriving at a stable interpretation.

As a core variable in language comprehension, semantic ambiguity refers to uncertainty in interpreting a linguistic unit, typically defined as the variability across possible interpretations. In classical information theory, uncertainty can be formalized as entropy, which provides a quantitative measure of uncertainty. Information is defined as a reduction in uncertainty (Shannon, 1948; MacKay, 2003; Venhuizen et al., 2019; Stone, 2022). Entropy has recently been applied to model predictive processing during sentence or discourse comprehension (Hale, 2001; S. L. Frank, 2013; Heilbron et al., 2022). However, its application in tracking changes in the semantic interpretation of ambiguous words remains rare. Given that disambiguation entails updating a distribution over candidate meanings, entropy provides a principled measure of how semantic uncertainty evolves during comprehension.

Multiple theoretical frameworks emphasize the central role of contextual information in semantic disambiguation. From the perspective of information theory, the interpretation of ambiguous words can be conceptualized as a process in which contextual input incrementally reduces semantic uncertainty until a single interpretation is reached. Probabilistic models suggest that comprehenders update their beliefs about word meaning in a Bayesian manner during sentence processing, starting with a prior probability distribution over possible meanings of a word, which is updated as new information from the context becomes available. The meaning with the highest posterior probability in the updated distribution is chosen as the interpretation of the word (Levy, 2008; M. C. Frank & Goodman, 2012). Other theories conceptualize semantic comprehension as a dynamic system in which meanings are situated in a multidimensional semantic space, with each potential interpretation corresponding to a “semantic attractor” (Elman, 2009; J. M. Rodd, 2020). This framework predicts that stronger contextual constraints facilitate faster and more reliable convergence toward the contextually appropriate interpretation (McClelland et al., 1989; J. M. Rodd, 2020). Parallel to this account, distributed semantic models also represent meaning as a continuous vector in semantic space (Landauer & Dumais, 1997; Mikolov et al., 2013), which is now a foundational principle in large language models (e.g., BERT; Devlin et al., 2019). These models compute contextualized word embeddings that support fine-grained semantic analysis and even partially align with the representational structure of the human language system (Toneva & Wehbe, 2019; Schrimpf et al., 2021; Yu et al., 2024; Xu et al., 2025).

Eye-tracking research has shown that gaze measures—such as first fixation duration, gaze duration, and regression probability—offer temporally fine-grained insights into how readers process lexical information during sentence comprehension. Classic studies (Duffy et al., 1988; Rayner, 2009) demonstrated that fixation times on ambiguous words are modulated by both lexical frequency and contextual bias, reflecting an interaction between bottom-up lexical properties and top-down contextual constraints. More recent work has incorporated probabilistic metrics, showing that fixation durations vary systematically with word predictability as estimated by surprisal (Smith & Levy, 2013; Staub, 2015), supporting an expectation-based view of real-time processing. Complementing this, Hoedemaker and Gordon (2014) showed that, even in a task-driven ocular lexical decision paradigm, gaze durations reveal robust semantic priming effects, suggesting that eye movements can serve as sensitive indicators of lexical–semantic activation under contextual guidance.

While prior studies confirm the role of context in guiding semantic interpretation, most empirical work has relied on static experimental designs comparing end-state interpretations across different contexts, using measures such as fixation duration (Duffy et al., 1988; Rayner, 2009; Smith & Levy, 2013; Staub, 2015) or reaction time (MacDonald et al., 1994; J. Rodd et al., 2002; Witzel & Forster, 2014). Such designs reveal the outcome of semantic selection but provide limited insight into how disambiguation unfolds in semantic space over time. Moreover, traditional behavioral measures offer only indirect evidence of semantic processing dynamics, lacking a direct index of uncertainty reduction. In addition, while models like BERT perform well on static disambiguation tasks (Huang et al., 2020; Zheng et al., 2021), it remains unclear whether they exhibit human-like sensitivity to the dynamic modulation of meaning by context—particularly in terms of how semantic uncertainty evolves over time and under varying levels of contextual constraint.

To address these issues, the present study combined a novel entropy-based measure of semantic uncertainty with time-resolved eye-tracking to investigate the dynamic evolution of semantic disambiguation during sentence comprehension. We recorded participants’ gaze patterns as they encountered ambiguous words embedded in sentences, which provided clues of how their interpretation of word meaning evolved over time. By analyzing the distribution of semantic choices across participants, we quantified uncertainty at the group level (i.e., aggregated across all participants) and assessed how this disambiguation process was modulated by contextual strength, semantic bias, and the timing of word presentation. Specifically, we designed a sentence-level eye-tracking experiment to examine how readers processed ambiguous words in real time within naturalistic linguistic contexts. Participants’ semantic preferences were collected, enabling an entropy-based quantification of semantic uncertainty under varying contextual conditions. In information theory, entropy provides a formal measure of uncertainty. In this study, it was employed to quantify the degree of semantic uncertainty, which captures the variability in word sense selection across different contextual conditions. This entropy approach allowed us to explore the mechanisms by which context influences the trajectory of semantic disambiguation, with a particular focus on how semantic bias modulates the rate of uncertainty reduction. Additionally, we applied the BERT model to the same set of stimuli and analyzed its internal representations to generate a model-based disambiguation trajectory. By comparing human and model data, we aimed to identify shared and divergent mechanisms in semantic processing and test theoretical predictions regarding the relationship between contextual constraints and the speed of semantic convergence.

## 2. Methods

### 2.1. Participants

Thirty-five native Chinese-speaking undergraduate or graduate students were recruited from Beijing Normal University. All of the participants read and signed an informed consent form and received appropriate remuneration after the experiment. All participants had normal or corrected-to-normal vision (visual acuity ≥ 1.0) and no reported history of neurological or language disorders. A total of 2 participants were excluded due to poor eye-tracking data quality (i.e., gaze loss rate > 30%), resulting in a final sample of 33 participants (7 males and 26 females; mean age = 23.06 years, SD = 2.73).

### 2.2. Apparatus

The experiment was programmed using PsychoPy (Version 2024.2.4, Peirce et al., 2019) and run on a Windows-based system. Visual stimuli were presented on a 72 cm × 45 cm LCD monitor, with a resolution of 2560 × 1440 pixels and a refresh rate of 100 Hz.

Eye movements were recorded using an EyeLink 1000 Plus system (SR Research) at a sampling rate of 1000 Hz. The EyeLink Developer’s Toolkit was used via Python (Version 3.8.19) for experimental control and data acquisition. A chin rest was used to stabilize the participant’s head and maintain a fixed viewing distance of 100 cm. Its height was adjusted to ensure that the participant’s eyes were level with three-quarters of the monitor’s vertical height.

### 2.3. Design

A within-subjects design was employed, with the semantic bias strength of the sentence context toward a particular meaning of the ambiguous word as the independent variable. The semantic bias levels were discretized into six levels, namely, [0, 0.2, 0.4, 0.6, 0.8, 1.0], where 0 indicates full bias toward Sense 1, 1.0 indicates full bias toward Sense 2, and the intermediate levels [0.2, 0.4, 0.6, 0.8] represent varying degrees of contextual support for either Sense 1 or Sense 2. A bias level of 0.5 reflects no bias toward either sense. Each participant completed trials across all bias conditions.

### 2.4. Stimuli

A total of 16 ambiguous Chinese words (11 nouns and 5 verbs) were selected, each associated with two distinct meanings, as defined in the SemEval-2007 Chinese dataset (Jin et al., 2007) (see Appendix A Table A1), with each word annotated with two sense entries. These entries may reflect either related senses (polysemous) or unrelated meanings (homonyms). Context sentences were constructed for each ambiguous word based on this dataset. For each of the 16 words, 66 sentences were created with controlled length. Thirty independent raters evaluated the semantic bias of each sentence using a five-point slider, assessing how much the context supported either Sense 1 or Sense 2. The ratings were used to establish six bias levels, reflecting varying degrees of support for each sense, from full bias toward Sense 1 (0) to full bias toward Sense 2 (1.0), with intermediate levels representing varying strengths of bias for each sense. Based on these ratings, 180 sentences (mean = 31 characters, SD < 4) were selected to span the six semantic bias levels. Each word was represented by at least one sentence per bias level; for eight of the words, two sentences were included per level (see Appendix A Table A2 for a representative example using the word “气息”). The majority of the sentences were declarative statements describing scenarios or facts, without dialogue or speaker-directed cues, providing consistent contexts primarily for lexical–semantic interpretation.

Audio versions of the sentences were generated using the Fish-speech AI synthesizer (https://github.com/fishaudio/fish-speech?tab=readme-ov-file, accessed on 19 January 2025), and 10 versions per sentence were produced with controlled speed and tone. Two linguistically trained raters selected the best version based on naturalness and rhythmic consistency. The audio was presented via Sennheiser HD280 Pro headphones.

Each trial involved a semantic selection task with four options, namely, two candidate meanings (as defined in SemEval-2007 and Modern Chinese Dictionary Sixth Edition) and two semantically related distractors, with all four option definitions limited to fewer than 10 characters. These distractors were meaningfully related to one of the possible senses of the ambiguous word, either from the same category or context. Specifically, one distractor was related to each candidate meaning. For words with highly similar meanings, both distractors were related to the core sense of the word. The distractors were chosen based on their semantic relevance to the ambiguous word’s senses, ensuring that they were contextually appropriate to a certain degree but distinct enough to increase task difficulty. This design enhanced semantic selection difficulty, introducing variability in responses and preventing the participants from fixating on the most obvious option.

**Figure 1:**
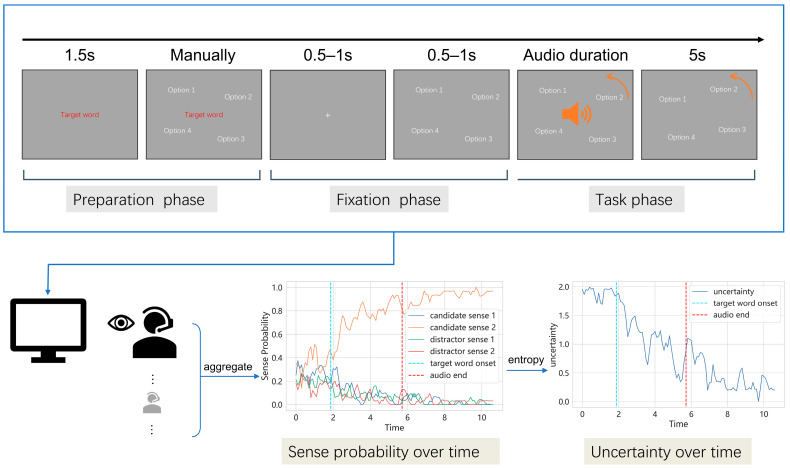
Schematic illustration of the stimulus presentation procedure in each trial. Participants listened to auditory stimuli through headphones while seated in front of a screen, responding by fixating on their chosen meaning of the ambiguous word. The meaning choices of all participants were aggregated to form a distribution of sense selections at each timepoint. Information entropy was then calculated over these distributions to estimate semantic uncertainty at each moment. The example time course of sense distribution and uncertainty shown are derived from the same representative trial.

The four meanings were presented on a circular layout centered on the screen, 10° from the center. The font height of each meaning was set to 1.2° of the visual angle. They were placed at four of five equidistant positions along the arc (see Figure 1), with random order and initial positions. During the task, the meanings rotated continuously at a constant speed of 5°/s in either a clockwise or counterclockwise direction (randomized per trial). This dynamic presentation was designed to (1) prevent the participants from fixating early on a specific location and (2) ensure a wider spatial distribution of gaze, which increases variability in gaze responses and allows for more comprehensive sampling across options. This variability helped track the evolving semantic focus and provided a more accurate reflection of the participants’ semantic interpretation process and uncertainty as they processed the context.

### 2.5. Procedure

The participants were instructed to minimize head movement to ensure accurate eye-tracking. The experiment consisted of 180 randomized experimental trials and 30 practice trials. The trials were divided into five blocks of 36 trials each, with self-paced breaks between the blocks.

Before the experiment, a calibration procedure was performed. A drift check was conducted before each trial, requiring an error less than 0.5°; otherwise, recalibration was initiated.

Each trial included the following stages:

- Preparation Phase: A red cue displaying the target ambiguous word appeared at the center of the screen. After 1.5 s, four option meanings were shown in a circular arrangement at a 10° radius (see Figure 1). This phase allowed the participants to recognize the target ambiguous word and become familiar with the option meanings and their spatial layout. The participants advanced the trial manually, at which point the option meanings and target ambiguous word disappeared.
- Fixation Phase: A fixation cross appeared for 0.5–1 s, followed by the reappearance of the four meanings.
- Task Phase: After a random delay (0.5–1 s), the auditory sentence began. Meanings started rotating at 5°/s in a randomized direction. The participants used their gaze to actively indicate the meaning that they currently believed best matched the sentence context, dynamically adjusting their gaze as the sentence unfolded. No feedback was given during formal trials (a red dot indicating gaze position was shown as feedback during practice). After the sentence ended, the rotating meanings remained visible for an additional 5 s to allow for final gaze-based responses. Then, the trial ended, and the participants entered a self-paced rest period.

No manual responses were required. The participants made semantic choices solely via gaze, which explicitly indicated the meaning that they selected based on the auditory context. This procedure allowed for the continuous tracking of their evolving semantic focus based on the auditory context, reflecting the uncertainty and variability in their semantic disambiguation process.

### 2.6. Data Preprocessing

Eye-tracking data were time-aligned to the sentence onset and extended 5 s beyond sentence offset to ensure that all task responses were captured. Data points were excluded if (1) they fell outside the screen area or (2) they occurred during saccades (defined as eye movements > 25°/s). Right-eye data were used by default; if missing, left-eye data were substituted.

### 2.7. Data Analysis

The analysis aimed to quantify the uncertainty in the participants’ semantic choices over time, thus revealing the contextual effects on dynamic word sense disambiguation.

When a gaze point was located near the area occupied by a specific option meaning, it was considered a selection of that meaning. To reduce the impact of drift, we defined rectangular bounding boxes around each option meaning, extending the width and height by one character size. Using gaze positions and bounding boxes, a time series of moment-by-moment meaning choices was computed for each trial. Overall, 92.18% of gaze points were successfully assigned to a specific option meaning, indicating that the participants’ attention was predominantly focused on the target areas throughout the trials.

Semantic uncertainty was calculated at the group level. Since gaze sampling points varied across participants and trials, a time binning method was used to standardize data. We divided each trial into fixed-length bins using 100 ms and 500 ms intervals (i.e., 10 Hz and 2 Hz sampling rates). Within each bin, the proportion of gaze samples assigned to each of the four meanings was computed (the number of selected option samples/total number of samples per bin).

For each time bin of each trial, all participants’ meaning selections were aggregated, yielding a probability distribution over meanings for each time bin. Based on these distributions, semantic uncertainty was quantified using Shannon entropy:

(1) $$H=-\sum_{i=1}^{n}p(x_{i})\log_{2}p\left(x_{i}\right),$$

where H represents Shannon entropy, and $p(x_{i})$ is the probability of selecting meaning *x* among the four meaning options. This entropy value reflects the degree of uncertainty or variability in meaning selection for the ambiguous word at each time bin within a given sentence trial. Higher entropy values indicate greater uncertainty. This resulted in time-resolved uncertainty profiles for each sentence trial (see Figure 1), available at both 100 ms and 500 ms resolutions.

To align the semantic uncertainty data with the sentence content, we used the CTC Forced Aligner (https://github.com/MahmoudAshraf97/ctc-forced-aligner, accessed on 19 January 2025) to align the sentence audio with the text, yielding word-level time stamps. Word-level onsets were used to align the uncertainty data with the sentence content, as the uncertainty data were captured at discrete timepoints, and our primary analysis was based on word-level semantic uncertainty.

## 3. Results

### 3.1. Comparison of Semantic Uncertainty Across Sentence Segments

To examine how cumulative linguistic input affects the semantic uncertainty of ambiguous words during sentence processing, we divided the task phase into three temporal segments: the initial segment (first half of the sentence), the late segment (second half of the sentence), and the post-sentence segment (5 s after sentence offset). Because the amount of linguistic information available to the participants differed across the three segments—with the later segments containing more information—this division allowed for an examination of the cumulative effects of semantic information during comprehension. For each segment, uncertainty values were averaged across all word-aligned timepoints from the time-resolved uncertainty profiles.

**Figure 2:**
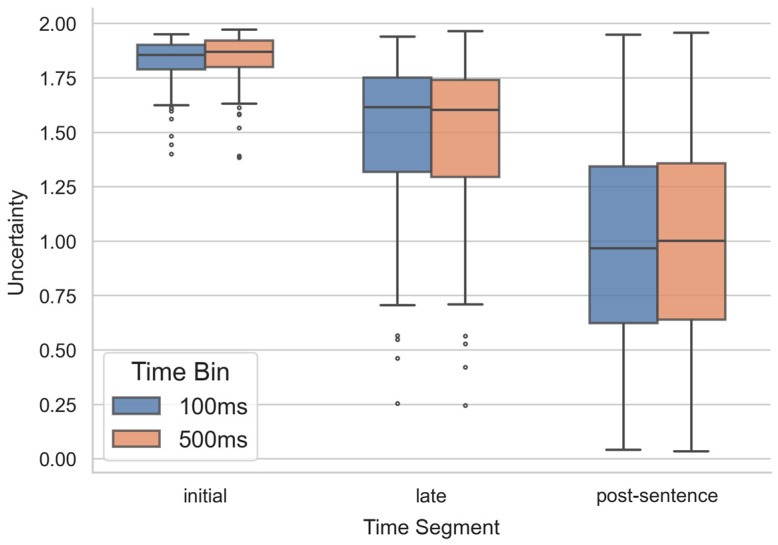
Semantic uncertainty across three sentence segments. Each trial was divided into initial (first half of the sentence), late (second half), and post-sentence phases (from sentence offset to trial end) based on sentence length. Uncertainty was consistently lower in later segments regardless of whether 100 ms or 500 ms time bins were used.

To examine the effect of the temporal segment, a repeated-measures ANOVA was conducted at the trial level. The results revealed a significant main effect of segment (*F*(2, 358) = 563.78, *p* < 0.001, η^2^ = 0.759). Pairwise Bonferroni-corrected *t*-tests showed that uncertainty was significantly lower in the late segment (M = 1.492, SD = 0.340) than in the initial segment (M = 1.828, SD = 0.100; *t*(179) = 15.92, *p* < 0.001, Cohen’s *d* = 1.186). The post-sentence segment (M = 0.972, SD = 0.454) also showed lower uncertainty than both the initial (*t*(179) = 26.71, *p* < 0.001, Cohen’s *d* = 1.991) and late segments (*t*(179) = 23.09, *p* < 0.001, Cohen’s *d* = 1.721). These results indicate that uncertainty declines significantly over time, with the reduction beginning even before sentence completion (see Figure 2). A comparable pattern was observed using 500 ms time bins; all subsequent analyses focused on 100 ms bins.

### 3.2. Effect of Ambiguous Word Appearance on Semantic Uncertainty

The position of an ambiguous word within a sentence may influence how comprehenders utilize and integrate contextual information, thereby affecting the semantic uncertainty of the ambiguous word. To test this hypothesis, we examined whether the appearance of the ambiguous word reduced semantic uncertainty. For each trial, uncertainty was averaged across the 1 s window before and after the ambiguous word onset. A paired *t*-test showed that pre-word uncertainty (M = 1.757, SD = 0.203) was significantly higher than post-word uncertainty (M = 1.599, SD = 0.309; *t*(179) = 11.63, *p* < 0.001, Cohen’s *d* = 0.87).

Although this effect indicates that uncertainty decreases following the onset of the ambiguous word, as shown in Section 3.1, uncertainty generally declines as contextual information accumulates. Therefore, the observed pre-/post-difference could simply reflect the overall reduction in uncertainty as more words are heard. To control for this global downward trend in uncertainty, we conducted a permutation test. Specifically, we sampled 10,000 random timepoints from each trial and computed a pre-/post-difference in mean uncertainty (1 s before vs. 1 s after) to construct a null distribution of t-values under the assumption of no event-specific effect. A one-sided comparison between the observed t-value and the null distribution showed that the observed ambiguity-related difference significantly exceeded the upper bound of this null distribution (*p* < 0.001). This result further supports the notion that the appearance of the ambiguous word has a unique and robust effect on semantic uncertainty, likely due to clarifying its grammatical role and facilitating semantic integration within the sentence.

### 3.3. Effects of Semantic Bias Strength

The semantic bias of context reflects the extent to which the context guides interpretation toward a particular meaning of an ambiguous word and may influence the speed of semantic convergence. To explore how semantic bias influences the rate of uncertainty reduction, a 3 (segment: initial, late, and post-sentence) × 6 (bias level: [0, 0.2, 0.4, 0.6, 0.8, 1.0]) repeated-measures ANOVA was performed. The results showed significant main effects of segment (*F*(2, 348) = 766.67, *p* < 0.001, η^2^ = 0.815) and bias (*F*(5, 174) = 10.70, *p* < 0.001, η^2^ = 0.235) and a significant interaction (*F*(10, 348) = 13.88, *p* < 0.001, η^2^ = 0.285).

**Figure 3:**
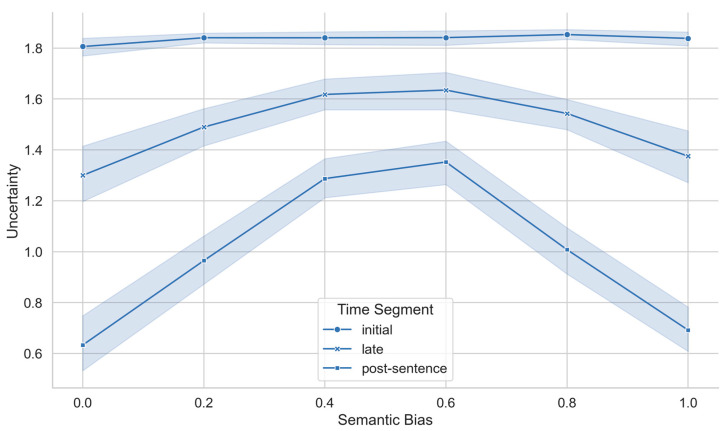
Semantic bias effects on uncertainty across three temporal segments. Bias levels 0.4, 0.2, and 0 reflect increasing contextual support for one meaning of the ambiguous word; levels 0.6, 0.8, and 1.0 reflect support for the alternative meaning. Uncertainty was modulated by semantic bias in the late and post-sentence segments.

Post hoc analyses revealed no effect of bias level in the initial segment but significant effects in the late (*F*(5, 174) = 4.61, *p* < 0.001, η^2^ = 0.117) and post-sentence segments (*F*(5, 174) = 18.01, *p* < 0.001, η^2^ = 0.341). Further analysis revealed that a stronger bias level was linked to lower uncertainty, with particularly pronounced differences between bias levels of 0 vs. 0.4 and 0.6 vs. 1.0 (see Figure 3). These results indicate that, while uncertainty did not differ by bias level in the initial segment, sentences with stronger contextual cues exhibited lower uncertainty in the late and post-sentence segments, suggesting that bias level accelerates the reduction in semantic uncertainty.

**Figure 4:**
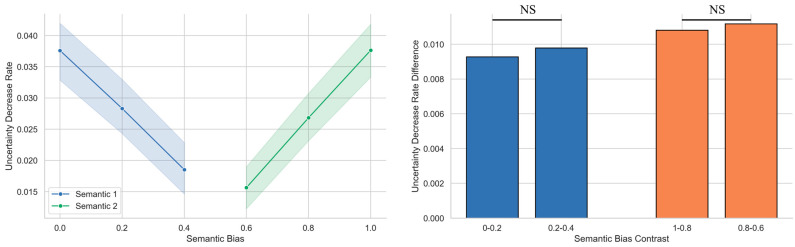
Semantic bias linearly modulates the rate of uncertainty reduction for ambiguous words. **Left panel:** Stronger semantic bias led to faster reductions in uncertainty. Bias levels 0.4, 0.2, and 0 reflect increasing support for one interpretation of the ambiguous word (semantic 1), while 0.6, 0.8, and 1.0 reflect increasing support for the alternative interpretation (semantic 2). **Right panel:** Equal increments in the semantic bias strength produced approximately equal changes in the uncertainty reduction rate regardless of the direction of bias.

To quantify the rate at which semantic uncertainty decreased during sentence processing, we first calculated the difference in uncertainty between the initial and post-sentence segments for each trial, and then we normalized this value by dividing it by the sentence length to obtain a standardized uncertainty reduction rate. A one-way ANOVA using the semantic bias level as the independent variable and the reduction rate as the dependent variable revealed a significant main effect (*F*(5, 174) = 18.23, *p* < 0.001, η^2^ = 0.344). Tukey’s HSD tests showed that the rates increased with a stronger bias, and the differences between adjacent levels were mostly significant (see the left panel of Figure 4). Specifically, the reduction rate under bias level 0 (M = 0.038, SE = 0.02) was significantly higher than that under 0.2 (M = 0.028, SE = 0.02; mean difference = 0.009, 95% CI [0.001, 0.018], *p* = 0.025, Cohen’s *d* = 0.732); 0.2 was higher than 0.4 (M = 0.019, SE = 0.02; mean difference = 0.010, 95% CI [0.001, 0.018], *p* = 0.017, Cohen’s *d* = 0.835); and bias level 0 was higher than 0.4 (mean difference = 0.019, 95% CI [0.010, 0.028], *p* < 0.001, Cohen’s *d* = 1.613). Similarly, bias level 1 (M = 0.038, SE = 0.02) was significantly higher than 0.8 (M = 0.027, SE = 0.02; mean difference = 0.011, 95% CI [0.002, 0.019], *p* = 0.004, Cohen’s *d* = 0.917); 0.8 was higher than 0.6 (M = 0.016, SE = 0.02; mean difference = 0.011, 95% CI [0.002, 0.020], *p* = 0.004, Cohen’s *d* = 1.081); and bias level 1 was also significantly higher than 0.6 (mean difference = 0.022, 95% CI [0.013, 0.031], *p* < 0.001, Cohen’s *d* = 2.014). These results suggest that a stronger semantic bias leads to a faster reduction in the semantic uncertainty of ambiguous words.

To determine whether these differences in reduction rates followed a linear pattern—i.e., whether equal steps in the semantic bias strength yielded equal increases in the convergence speed—we computed second-order differences (ΔM). Specifically, we compared the difference between bias levels 0 and 0.2 with that between 0.2 and 0.4 (ΔM1) and the difference between 1 and 0.8 with that between 0.8 and 0.6 (ΔM2) (see the right panel of Figure 4). Bootstrap analysis (10,000 iterations) showed the following: ΔM1 = 0.0005, 95% CI [–0.0098, 0.0111], *p* = 0.922; ΔM2 = 0.0004, 95% CI [–0.0091, 0.0098], *p* = 0.941. Neither was significant, supporting the interpretation that uncertainty reduction rates increased approximately linearly with semantic bias.

Since the convergence rate could be influenced by the onset of disambiguation, we used piecewise linear regression to identify the period of the fastest uncertainty decline in each trial. The onset time of this segment was labeled as the “turning point,” and the slope within that segment was used as a refined index of the reduction speed. Two separate one-way ANOVAs showed that the bias strength had no effect on turning point timing (*F*(5, 174) = 0.203, *p* = 0.961) but significantly affected the slope (*F*(5, 174) = 3.128, *p* = 0.010, η^2^ = 0.082). These findings indicate that semantic bias primarily modulates the speed of disambiguation rather than the specific moment at which it begins.

### 3.4. Comparison with BERT’s Semantic Uncertainty

Human semantic uncertainty—quantified as the entropy of sense selection based on eye-tracking responses—has been shown to be dynamically modulated by sentence context. To assess whether BERT exhibits a comparable sensitivity to context when computing the meaning of ambiguous words, we analyzed the dynamic uncertainty profiles generated by a pretrained Chinese BERT model (RoBERTa-wwm-ext, Cui et al., 2020) and compared them with the human-derived uncertainty series.

We defined BERT’s uncertainty at each timepoint as the relative distance between the ambiguous word’s representation and two contextually influenced reference sense embeddings. These reference sense embeddings were derived by averaging the BERT embeddings of sentences from the SemEval-2007-annotated corpus, where each sense of the ambiguous word was annotated. The relative distance was calculated by projecting the ambiguous word’s embedding onto the difference vector between the two reference sense embeddings and normalizing the distance along this vector. When the representation was equidistant from both senses, uncertainty was defined as maximal (1). When it coincided with one of the senses, uncertainty was minimal (0). Other intermediate values were linearly scaled based on the proximity to the nearest reference sense. These contextualized representations were extracted from the final hidden layer of the model. At each timepoint, the sentence was truncated to the portion presented up to that point, with all remaining characters masked (using BERT’s [MASK] token), except for the ambiguous word. This yielded a full dynamic sequence of BERT uncertainty values for each of the 180 experimental sentences.

To evaluate the overall similarity between the BERT-derived and human-derived uncertainty, we concatenated the trial-level uncertainty sequences into two vectors and computed a Pearson correlation. The result showed a significant positive correlation (*r*(178) = 0.170, *p* < 0.001), indicating shared global trends.

**Figure 5:**
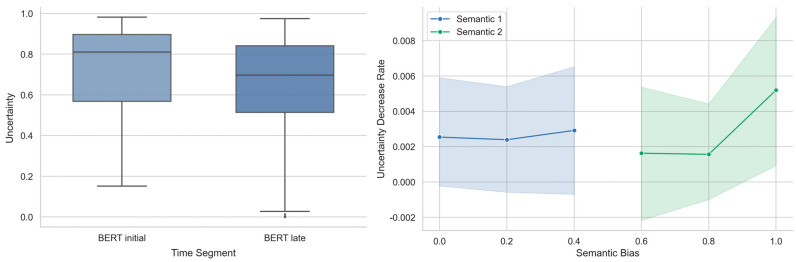
BERT’s semantic uncertainty for ambiguous words decreased over time but was not modulated by the semantic bias strength. **Left panel:** The sentence was divided into two halves based on length—initial (first half) and late (second half). Uncertainty was lower in the late segment than in the initial segment, indicating a general downward trend. **Right panel:** Semantic bias did not significantly influence BERT’s rate of uncertainty reduction regardless of the direction of bias. Bias levels 0.4, 0.2, and 0 indicate increasing contextual support for one interpretation of the ambiguous word (semantic 1), while 0.6, 0.8, and 1.0 indicate support for the alternative interpretation (semantic 2).

To further examine the source of this similarity, we performed analyses on BERT uncertainty analogous to those conducted on human data. First, we compared BERT uncertainty across the sentence segments. A paired *t*-test revealed that uncertainty was significantly higher in the initial segment (M = 0.739, SE = 0.014) than in the late segment (M = 0.650, SE = 0.018; *t*(179) = 4.363, *p* < 0.001, Cohen’s *d* = 0.917), mirroring the human pattern (see the left panel of Figure 5).

Next, we computed the rate of uncertainty reduction for BERT. Unlike human trials, BERT provides no post-sentence continuation time. Therefore, the reduction rate was computed by subtracting the average uncertainty during the initial segment from the final uncertainty value (i.e., after processing the complete sentence) and dividing the result by the sentence length. A one-way ANOVA using the semantic bias level as a predictor showed no significant effect (*F*(5, 174) = 0.580, *p* = 0.716, η^2^ = 0.016), diverging from the human results (see the right panel of Figure 5).

In summary, while BERT and humans showed similar trends in uncertainty reduction over time, they differ in how semantic bias modulates this reduction over time. For humans, the rate of uncertainty reduction accelerated as contextual bias increased; in contrast, BERT’s disambiguation rate remained largely unaffected by the degree of contextual bias.

## 4. Discussion

Previous research has rarely addressed the dynamic evolution of semantic disambiguation during incremental sentence comprehension, especially regarding changes in semantic uncertainty over time. The present study used eye-tracking to trace trial-by-trial distributions of sense selections when participants encountered ambiguous words within sentence contexts. This enabled the reconstruction of the temporal trajectory of semantic uncertainty, offering a real-time depiction of the disambiguation process. Moreover, we manipulated the strength of contextual semantic bias to examine how it modulates disambiguation dynamics.

The results revealed that human semantic uncertainty is influenced by both the accumulation of contextual information and the timing of the ambiguous word’s appearance within the sentence. While BERT similarly demonstrated a decline in semantic uncertainty as the amount of contextual information increased, it failed to show sensitivity to variations in the semantic bias strength, unlike the human participants. Additionally, our findings indicate that, for humans, the reduction in uncertainty is gradual rather than abrupt, suggesting a progressive integration of semantic cues rather than a binary switch between interpretations.

A key methodological challenge in measuring semantic preference lies in the inadequacy of single-choice tasks to quantify the degree of preference for competing senses. However, repeated exposure to the same stimuli within participants may lead to carryover effects—including practice, adaptation, or fatigue—which interfere with the reliability of semantic preference measures (Greenwald, 1976). In contrast, aggregating responses across participants presented with the same sentence leverages their shared linguistic experiences and internal semantic models to yield representative group-level choice distributions. The high temporal resolution of eye-tracking, combined with time-windowed sampling, allowed us to obtain repeated semantic preference measurements for the same participant within short periods, thereby enhancing measurement stability.

This method produced clear sense selection trajectories, capturing increases, turning points, and reductions in preference strength. The entropy of these distributions effectively reflected changes in disambiguation states and responded sensitively to experimental manipulations. We observed a significant decline in semantic uncertainty as sentences unfolded, reflecting a gradual convergence of semantic representations under contextual guidance. Crucially, the appearance of the ambiguous word itself triggered a marked drop in uncertainty, likely because it unveiled additional syntactic cues, allowing the participants to better integrate the ambiguous word into the sentence and infer its intended meaning. These findings underscore the importance of contextual richness in facilitating lexical disambiguation. This aligns with prior research showing that semantically constraining contexts aid ambiguity resolution. For instance, Duffy et al. (1988) demonstrated that biased contexts reduce gaze durations on ambiguous words. In studies combining language and vision, Tanenhaus et al. (1995) and Kamide et al. (2003) showed that listeners use visual scene cues to rapidly narrow down lexical interpretations.

More intriguingly, we found that the rate of uncertainty reduction was systematically modulated by the semantic bias strength, following an approximately linear trend. Since uncertainty is derived from underlying semantic representations, this result indicates that the speed of semantic convergence is linearly regulated by contextual cues. This is consistent with several theoretical models. First, dynamic accounts of semantic representation conceptualize meaning comprehension as a vectorial convergence process in a high-dimensional semantic space, where contextual forces guide the system toward a target sense. The strength of contextual bias determines the convergence trajectory and rate—stronger cues accelerate convergence (Elman, 2009; J. M. Rodd, 2020). Second, constraint satisfaction models argue that ambiguity resolution arises from the dynamic competition between constraints at multiple levels—lexical, syntactic, and pragmatic. A greater and more consistent constraint strength yields faster resolution (MacDonald, 1994). While prior studies have provided indirect support for these models through behavioral (e.g., reading times) or neural (e.g., ERP amplitudes) indices (Federmeier, 2007), few have directly tested them using graded semantic bias manipulation and real-time uncertainty tracking. Our study addresses this gap by combining fine-grained bias manipulation with a dynamic measurement of disambiguation, providing direct empirical support for these theories.

Modern large language models (LLMs), such as BERT, employ distributed semantic representations in vector spaces and have been used to model human language comprehension. Prior studies have shown that LLMs can predict neural responses to linguistic stimuli (Goldstein et al., 2022; Schmitt et al., 2021), though key differences remain. For instance, Caucheteux et al. (2023) found that brain-based semantic representations exhibit long-range predictive dynamics, whereas LLMs primarily rely on short-range contexts. Xu et al. (2025) further reported that the representational similarity between models and brains drops significantly in sensorimotor domains, revealing structural divergences in semantic systems. Our findings add to this literature: although BERT captured the general trend of decreasing uncertainty with accumulating context, it failed to replicate the human-like modulation of disambiguation speed by semantic bias. This discrepancy may arise from differences in the semantic architecture. The human system may operate via an attractor-based mechanism, where representations rapidly converge once sufficient evidence accumulates (Plaut et al., 1996; McLeod et al., 2000; J. M. Rodd, 2020). In contrast, BERT’s representational dynamics are relatively static, lacking flexible adjustment to input variation. Consequently, the model’s semantic updates under different bias conditions remain limited. These findings suggest that future efforts to model human-like semantic processing may require incorporating more dynamic mechanisms into representational architectures.

Our experimental design deliberately incorporated variability through the dynamic rotation of option meanings, which broadened the range of gaze responses and enabled more comprehensive sampling across multiple options. The selection of semantically related distractors, meaningfully connected to one of the candidate meanings, further amplified this variability. Although gaze data can be influenced by factors beyond semantic interpretation, such as subconscious orienting, this variability aligned with our objective of measuring semantic uncertainty. Instead of enforcing a deterministic interpretation, the design embraced the probabilistic nature of semantic understanding by continuously tracking the participants’ gaze across multiple meanings, providing insights into the uncertainty in their interpretation.

It should be emphasized that gaze does not directly reflect participants’ intended responses, but rather it tracks their evolving semantic focus, serving as an indirect indicator of meaning interpretation. By aggregating gaze data at the group level, we derived a dynamic measure of semantic understanding that tracks the evolving disambiguation process over time. This method provides a nuanced depiction of how uncertainty is resolved in context.

Despite the valuable insights provided by this study, there are several limitations that should be considered. One limitation of the present study is that semantic uncertainty was estimated from group-level distributions, which may obscure individual variability in disambiguation dynamics. Additionally, our comparison focused on BERT, a commonly used and well-characterized model for semantic disambiguation tasks. While this allowed for a controlled contrast with human data, future research could expand the model space. For instance, newer generative models aligned with human dialogue behavior—such as OPT (Zhang et al., 2022), LLaMA (Touvron et al., 2023), or Alpaca (Taori et al., 2023)—may exhibit different disambiguation patterns according to context. Investigating how various architectures handle contextual ambiguity could further clarify the boundary conditions for human–model alignment in semantic processing.

## 5. Conclusions

In conclusion, by leveraging group-based dynamic semantic selection, we traced the real-time evolution of human semantic uncertainty as sentences unfolded. The results show that semantic uncertainty declined over time and that the rate of decline scaled linearly with the contextual bias strength. In contrast to human participants, while BERT captured the overall reduction trend, it lacked the human-like flexibility in context-sensitive modulation. These findings highlight the current limitations of LLMs in modeling the nuanced aspects of human semantic understanding.

## Data Availability

The raw data supporting the conclusions of this article will be made available by the authors on request.

## Appendix A

**Table A1:** The table lists each ambiguous word in Chinese and its two possible interpretations in English (Sense 1 and Sense 2), as defined in SemEval-2007.

Ambiguous Word (Chinese)	Sense 1 (English)	Sense 2 (English)
中医	traditional Chinese medical science	practitioner of Chinese medicine
儿女	young man and woman	children
单位	organization	unit
机组	aircrew	set of machines
气息	spirit	smell
气象	meteorology	atmosphere
牌子	brand	plate
眼光	foresight	eye
菜	vegetable	dish
表面	appearance	face
镜头	shot	lens
动摇	shake	vacillate
平息	put down	subside
推翻	repudiate	overthrow
说明	show	explain
震惊	shock	be shocked

**Table A2:** Examples of sentences for the ambiguous word “气息” across different semantic bias levels. The table presents original Chinese sentences and their English translations. In each English example, the word location of “气息” is aligned with its Chinese sense to ensure coherence with the sentence context. This appendix illustrates how “气息” serves as a representative ambiguous word whose meaning shifts gradually from spirit to smell along the semantic bias continuum.

Semantic Bias Level	Sentence Sample (Chinese)	Sentence Sample (English)
0	那幅画展现了深厚的文化底蕴，每一笔都散发着让人陶醉不已的气息。	The painting showcases profound cultural heritage, and every brushstroke exudes an intoxicating (气息: spirit).
0.2	这个老式图书馆内的气息，让人仿佛穿越回过去，每一本书都是一个时代的见证。	The (气息: spirit) in this old-fashioned library makes one feel as if stepping back in time, with every book witnessing an era.
0.4	春天的花园里，每一片叶子、每一个角落都散发着生机勃勃的气息。	In the spring garden, every leaf and every corner radiates vibrant (气息: spirit).
0.6	即使在繁忙的都市中心，那隐约透出自然气息的小花园也能带给人片刻宁静。	Even in the bustling city center, the small garden with a hint of natural (气息: smell) brings a moment of calm.
0.8	气息渐浓，夜幕下的公园里传来阵阵烧烤的香味，人们在灯光下欢声笑语。	The (气息: smell) grows stronger; in the park under the night sky, the smell of barbecue drifts, and people laugh joyfully under the lights.
1	春天的到来，校园中弥漫着花草的气息，学生们笑声朗朗。	With the arrival of spring, the campus is filled with the (气息: smell) of flowers and plants, accompanied by the students’ cheerful laughter.
